# Recommendations for breastfeeding during Coronavirus Disease 2019 (COVID-19) pandemic

**DOI:** 10.1186/s13006-022-00465-w

**Published:** 2022-04-11

**Authors:** Xiyao Liu, Haoyue Chen, Meijing An, Wangxing Yang, Yujie Wen, Zhihuan Cai, Lulu Wang, Qianling Zhou

**Affiliations:** Department of Maternal and Child Health, School of Public Health, Peking University, No. 38 Xueyuan Road, Haidian District, Beijing, 100191 China

**Keywords:** COVID-19, SARS-CoV-2, Breastfeeding, Safety, Recommendation, Review

## Abstract

**Background:**

Coronavirus Disease 2019 (COVID-19) has spread worldwide. The safety of breastfeeding of SARS-CoV-2-positive women has not yet reached a consensus among the scientific community, healthcare providers, experts in lactation care, health organizations and governments. This study was conducted to summarize the latest evidence about the safety of breastfeeding among suspected/confirmed infected mothers and to summarize the recommendations on breastfeeding during COVID-19 from different organizations.

**Methods:**

A comprehensive literature review of publications about the safety of breastfeeding among SARS-CoV-2-infected mothers was conducted. Scientific databases were searched up to 26 May 2021. The evidence was summarized into five perspectives according to a framework proposed by van de Perre et al. with certain modifications. Moreover, websites of different health organizations were visited to gather the recommendations for breastfeeding.

**Results:**

The current evidence demonstrated that the majority of infants breastfed by infected mothers were negative for SARS-CoV-2. Breast milk samples from suspected/infected mothers mainly demonstrated negative results in SARS-CoV-2 viral tests. There was insufficient evidence proving the infectivity of breast milk from infected mothers. Recent studies found other transmission modalities (e.g., milk containers, skin) associated with breastfeeding. Specific antibodies in the breast milk of infected mothers were also found, implying protective effects for their breastfed children. According to van de Perre’s criteria, the breast milk of infected mothers was unlikely to transmit SARS-CoV-2. Owing to the low quality of the current evidence, studies with a more robust design are needed to strengthen the conclusion regarding the safety of breastfeeding. Further studies to follow up the health status of infants who were directly breastfed by their suspected/infected mothers, to collect breast milk samples at multiple time points for viral tests and to examine specific antibodies in breast milk samples are warranted. Current recommendations on breastfeeding during COVID-19 from different organizations are controversial, while direct breastfeeding with contact precautions is generally suggested as the first choice for infected mothers.

**Conclusions:**

This review determined the safety of breastfeeding and identified the focus for further research during the COVID-19 pandemic. Recommendations on breastfeeding are suggested to be updated in a timely manner according to the latest evidence.

## Background

Coronavirus Disease 2019 (COVID-19) is a viral infection caused by a novel coronavirus named Severe Acute Respiratory Syndrome Coronavirus 2 (SARS-CoV-2) [1]. COVID-19 was characterized as a global pandemic by the World Health Organization (WHO) in March 2020 [2]. To date, COVID-19 is still epidemic in most areas of the world, such as Europe, the Americas, and Southeast Asia [3]. The pandemic is more serious in the Americas (contributing to 38.2% of cases and 46.1% of deaths) than in other areas. The North American region accounted for the highest proportions of cases (76%) and deaths (72%). The number of confirmed infected cases was 243,327,429 globally as of 23 October 2021, including 4,943,742 (2.03%) deaths [4].

Pregnant women and young children are susceptible to COVID-19 [5, 6]. During pregnancy, some adaptive immune responses in pregnant women are downregulated (e.g., the decrease in the number of T cells and B cells) [7]. Additionally, the upper respiratory tract tends to be swollen due to high levels of estrogen and progesterone, and restricted lung expansion makes pregnant women susceptible to respiratory pathogens [5]. As newborns do not have antibodies against coronaviruses, they are theoretically more vulnerable to SARS-CoV-2 infection [6]. Therefore, lactation among infected mothers deserves special attention during this pandemic. Although the benefits of breastfeeding for mothers and children have been well acknowledged [8], SARS-CoV-2-positive women are still concerned about the risks of virus transmission from mother to infant during breastfeeding [9]. There were two reviews on breastfeeding and COVID-19 published in 2020. Both recommended breastfeeding among infected mothers [10, 11]. However, much new evidence emerged in the following year. A comprehensive summary of the current evidence verifying the safety of breastfeeding among SARS-CoV-2-positive women is still needed. Moreover, recommendations put forwards by different national authorities and health organizations have been updated [8, 10, 12–16]. Evidence on the safety of breast milk from SARS-CoV-2-infected mothers and recommendations of breastfeeding practices that have not yet reached a consensus may lead to anxiety and affect the health and survival of young children.

To determine the plausibility of viral transmission by breast milk, van de Perre et al. [17] established an analytical framework using the underlying principles of Koch’s postulate. The framework was proposed to help clarify the relationship between breastfeeding exposure and viral infections and was based on five criteria: viral infection in children breastfed by infected mothers; the presence of virus/antigen/genome in the breast milk of infected mothers; the infectivity of virus in breast milk; attempts to rule out other transmission modalities; and the reproduction of transmission by breast milk in an animal model. If five criteria were met, there was 100% possibility of virus transmission. If four criteria were met, virus transmission was very likely to occur. If three criteria were met, virus transmission was possible. If two criteria and even fewer were met, virus transmission was unlikely [17].

The present review was conducted to summarize 1) the latest evidence about the safety of breastfeeding among SARS-CoV-2-infected mothers and 2) the recommendations on breastfeeding from different organizations during the COVID-19 pandemic. The analytical framework by van de Perre et al. [17] was adopted in the present review to summarize the existing evidence. In the current literature, animal studies about breastfeeding and SARS-CoV-2 transmission were not available. However, there have been studies reporting SARS-CoV-2-specific antibodies in the breast milk of infected mothers. As a result, modification of van de Perre’s framework [17] was made in the present review. This review might be useful to ensure optimal infant feeding practices, as well as maternal and child health over the critical period of the COVID-19 pandemic.

## Methods

### Search strategy and selection criteria

A comprehensive literature review of the publications on breastfeeding during COVID-19 to date was conducted. The scope of the literature search included databases of journal articles and official websites of the health organizations. Due to the authors’ language literacy, articles and recommendations written in English and Chinese were included.

Databases including PubMed, Scopus, Embase, Web of Science, Cochrane Library, China National Knowledge Infrastructure (CNKI), and WANFANG DATA were searched up to 26 May 2021 regarding the evidence about the safety of breastfeeding among SARS-CoV-2-infected mothers. The key words for searching included “COVID-19”, “SARS-CoV-2”, “breastfeeding”, “formula feeding”, “breast milk”, “human milk”, “antibodies”, “antiviral”, “pregnant”, “infant”, “neonate”, and “newborn”. The reference lists of retrieved reviews were also manually searched. Journal articles, including those published online ahead of print, were included. The inclusion criteria were as follows: (1) the subjects were lactating mothers diagnosed with COVID-19 or SARS-CoV-2 infection; and (2) the study outcome was the safety of breast milk and/or the wellbeing of infants. The exclusion criteria were (1) conference abstracts, preprints, comments, and letters; (2) studies not published in Chinese or English; (3) studies for which the full text was not retrievable; (4) irrelevant studies; and (5) incomplete studies or studies without outcome information. Two researchers (XL and HC) independently searched, screened, and reviewed the literature. Disagreements were resolved by consulting a third researcher (MA). The evidence included in the present review was then summarized into five perspectives according to van de Perre’s framework [17] with certain modifications.

For recommendations, official websites of medical institutions and governmental and nongovernmental organizations were searched, including the American Academy of Pediatrics (AAP), Academy of Breastfeeding Medicine (ABM), U.S. Centers for Disease Control and Prevention (CDC), Italian National Institute of Health (ISS), International Society of Ultrasound in Obstetrics and Gynecology (ISUOG), National Health Commission of the People’s Republic of China, Royal College of Obstetricians and Gynecologists (RCOG), United Nations International Children’s Emergency Fund (UNICEF), and WHO. The recommendations were then summarized into a table.

## Results

### Evidence about the safety of breastfeeding

**Fig. 1:**
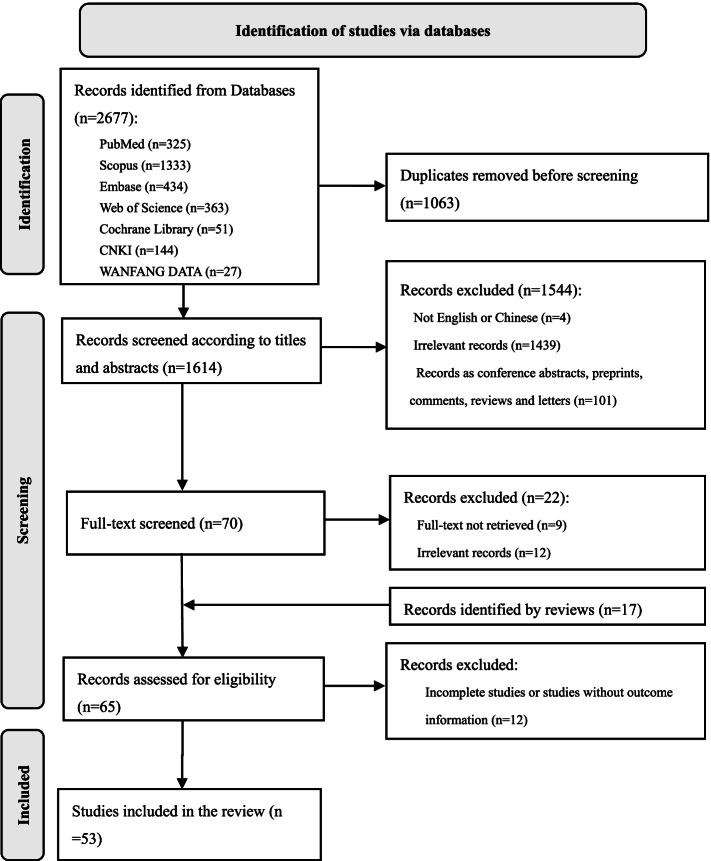
Flowchart of the study selection

A total of 2677 articles were identified by the comprehensive literature search. After excluding duplicates, the titles, abstracts and full texts were screened. A total of 53 articles were included in this review (Fig. 1. Flowchart of the study selection). Of the included studies, 16 reported the infection status of infants who had been breastfed by infected mothers; 33 reported the results of SARS-CoV-2 detection in breast milk from infected mothers; five were associated with the infectivity of breast milk from infected mothers; four assessed other relevant transmission modalities associated with breastfeeding; and nine were associated with the protective effect of breast milk. The included studies were summarized into the following five perspectives according to the framework mentioned above [17].

### Infection status of infants breastfed by infected mothers

Breastfed infants of SARS-CoV-2-positive mothers were followed-up with health assessments (Table 1). Among 16 studies included in this perspective, six found that breastfed children were positive for SARS-CoV-2. Notably, some breastfed infants who were positive for SARS-CoV-2 did not show clinical symptoms and turned negative several days later. In Italy [24], a preterm newborn was inadvertently fed with SARS-CoV-2-positive expressed breast milk. However, this newborn was not infected. Another study in Italy [6] showed that two breastfed infants tested SARS-CoV-2 positive 3 days and 7 days postpartum, respectively. However, both infants turned negative on the 14th day after delivery.

**Table 1:** Summary of evidence on breastfeeding and infant outcomes

Authors	Study site	Design	Follow-up time	Breastfeeding			No breastfeeding	
				The number of infants	Exclusive breastfeeding or not	Outcome ^**a**^	The number of infants	Outcome ^**a**^
Ajith et al. [18]	India	Single-center observational study	Within 24 h of delivery	165	NA	31 positive	56	1 positive
Bertino et al. [19]	Italy	Prospective observational study	48 h and 6 weeks postpartum	13	Yes	4 positive in the first 48 h of life; becoming negative by 6 weeks of life	1	Negative
Biasucci et al. [6]	Italy	Prospective study	3, 7 and 14 days postpartum	13	NA	1 positive on 3 days postpartum 1 positive on 7 days postpartum All negative on 14 days postpartum	2	All negative
Chu et al. [20]	China	Case report	1 month postpartum	1	NA	Negative	0	–
Cojocaru et al. [21]	USA	Retrospective study	24 h, 48 h and a week postpartum during admission	16	NA	All negative	15	All negative
Gao et al. [22]	China	Ambispective observational clinical analysis	During admission	4	NA	All negative	10	All negative
Kilic et al. [23]	Turkey	Prospective observational study	During the 14-day isolation	12	NA	6 positive	3	2 positive
Lugli et al. [24]	Italy	Case report	8, 10 and 18 days postpartum	1	NA	Negative	0	–
Oncel et al. [25]	Turkey	Multicenter cohort study	1, 2, 5 days postpartum	54	NA	All negative	71	4 positive
Pereira et al. [26]	Spain	Retrospective case series study	1.8 months	17	15 exclusive breastfeeding and 2 supplementing with formula	All negative	3	All negative
Piersigilli et al. [27]	Belgium	Case report	7 and 14 days postpartum	1	NA	7-day postpartum positive and14-day negative	0	–
Salvatore et al. [28]	USA	Cohort study	5–7 days and 14 days postpartum	64	With or without addition of formula	All negative	18	All negative
Savasi et al. [29]	Italy	Prospective multicenter cohort study	Early postpartum period	57	NA	4 positive	0	–
Shlomai et al. [30]	Israel	Multicenter study	14 to 21 days post-discharge	47	Yes	All negative	8	All negative
Tran et al. [31]	Vietnam	Case report	34 days postpartum	1	Yes	Negative	0	–
Vila-Candel et al. [32]	Spain	Retrospective and multicenter study	During admission	10	Yes	All negative	3	All negative

-: There was no infant who was not breastfed included in the study

^a^Some studies failed to obtain follow-up SARS-CoV-2 detection results of all infants

### The likelihood of SARS-CoV-2 contained in breast milk

Thirty-three studies included in this review examined whether SARS-CoV-2 existed in breast milk samples collected from mothers with COVID-19 during pregnancy or lactation (Table 2). The RT-PCR method was conducted in all included studies to detect SARS-CoV-2 nucleic acid in breast milk samples.

**Table 2:** Summary of evidence about SARS-CoV-2 examination in breast milk samples

Authors	Study country	Study design	Sample size	Time of mothers’ SARS-CoV-2 positive test	Time points of breast milk collection	RT–PCR test results
Bastug et al. [33]	Turkey	Case report	1	On the day of delivery	On the day of delivery and the 3rd, 4th day after delivery	3 positive
Bertino et al. [19]	Italy	Prospective collaborative observational study	14	Several days before or after delivery, 0–12 days before breast milk collection	Several times after delivery	1 positive
Buonsenso et al. [34]	Italy	Observational study	2	5, 7 days before delivery, respectively	Mother 1: on the 11th and 14th day after delivery; Mother 2: during the first 5 days after delivery	3 out of 5 samples from Mother 2 were positive
Chambers et al. [35]	USA	Case series	18	After delivery	A total of 64 milk samples were collected at different time points before and after the positive SARS-CoV-2 test result.	1 positive collected on the day of symptom onset
Chen et al. [36]	China	Case series	6	Third trimester of pregnancy	After first lactation	All negative
Chu et al. [20]	China	Case report	1	22 days after delivery	9 and 10 days after mother’s SARS-CoV-2 positive test	All negative
Cui et al. [37]	China	Case report	1	More than one month after delivery	2–4 days after mother’s SARS-CoV-2 positive test	All negative
Dong et al. [38]	China	Case report	1	22 days before delivery	6 days after delivery	Negative
Fan et al. [39]	China	Case report	2	4, 5 days before delivery, respectively	After delivery	All negative
Fenizia et al. [40]	Italy	Prospective multicenter study	11	Before delivery	5 days after delivery	1 positive
Gao et al. [22]	China	Ambispective observational clinical analysis	12	Before delivery	Within 7 days after delivery	All negative
Han et al. [41]	Korea	Case report	1	After delivery	After mother’s SARS-CoV-2 positive test	Negative
Hinojosa-Velasco et al. [42]	Mexico	Case report	1	On the day of delivery	Collected on the fourth day after delivery	Positive
Kalafat et al. [43]	Turkey	Case report	1	On the day of delivery	After mother’s SARS-CoV-2 positive test	Negative
Kam et al. [44]	Singapore	Case report	1	6 month after delivery	10 days after mother’s SARS-CoV-2 positive test	Negative
Kilic et al. [23]	Turkey	Prospective observational study	15	Lactation period	A total of 26 milk samples were collected within 2 days after the mothers’ symptoms began	4 positive
Lei et al. [45]	China	Case series	4	Several days before or after delivery	After mothers’ SARS-CoV-2 positive test	All negative
Liu et al. [46]	China	Case series	10	During late pregnancy	After first lactation	All negative
Lugli et al. [24]	Italy	Case report	1	9 days after delivery	Before or after mothers’ SARS-CoV-2 positive test	All positive
Marin Gabriel et al. [47]	Spain	Observational prospective study	7	6 on the day of delivery; 1 two months before delivery	Within the first hour after delivery	All negative
Mattar et al. [48]	Singapore	Prospective observational study	2	50, 81 days before delivery, respectively	Colostrum samples	All negative
Pace et al. [49]	USA	Prospective study	18	6.8 ± 7.8 months after delivery	A total of 37 milk samples were collected after mothers’ SARS-CoV-2 positive test	All negative
Peng et al. [50]	China	Longitudinal study	16	Before delivery	A total of 44 milk samples were collected on the day of delivery, the 3rd, 7th, 14th, 21st, 28th, 35th, 42nd, 56th and 70th day after delivery	All negative
Peng et al. [51]	China	Case report	1	One day before delivery	At day 2, 3, 4, 5, 6, 7, 10 and 14 of delivery	All negative
Piersigilli et al. [27]	Belgium	Case report	1	7 days after delivery	Before mothers’ SARS-CoV-2 positive test	Negative
Sharma et al. [52]	India	Ambispective observational study	23	Second or third trimester of delivery	After mothers’ SARS-CoV-2 positive test	All negative
Tam et al. [53]	Australia	Case report	1	8 months after delivery	A total of 7 milk samples were collected between 6 to 16 days after mothers’ SARS-CoV-2 positive test	2 positive
Thanigainathan et al. [54]	India	Descriptive study	30	Before delivery	Between 48 to 72 h after delivery	1 positive
Wang et al. [55]	China	Case report	1	On the day of delivery	36 h after delivery	Negative
Wu et al. [56]	China	Case series	3	The last month of pregnancy	On the 1st, 6th and 27th day after delivery	1 positive (collected 1st day after delivery)
Xiong et al. [57]	China	Case report	1	37 days before delivery	At the day of delivery	Negative
Yu et al. [58]	China	Case report	1	More than one year after delivery	On the 2nd, 9th, 16th and 19th day after mothers’ SARS-CoV-2 positive test	All negative
Zhuang et al. [59]	China	Case report	1	1 day after delivery	On the 5th day after delivery	Negative

There were 14 studies examining breast milk at a single time point and 14 studies examining breast milk at multiple time points. The majority of evidence demonstrated SARS-CoV-2 negative results in breast milk. For example, a study in Spain examined hand-expressed colostrum samples from seven infected women within the first hour of delivery; all seven breast milk samples tested SARS-CoV-2 negative [47]. Similarly, in China, two separate studies (*n* = 6 [36] and *n* = 10 [46]) reported that breast milk samples collected from infected mothers during their first lactation were found to be negative for SARS-CoV-2. Another two studies in China also reported negative results of breast milk samples collected from infected mothers at 36 h [55] and the sixth day [38] postdelivery. There were also examples of studies that conducted assessments at multiple time points demonstrating negative results. A study from China [58] reported that a mother’s nasopharyngeal swab specimens collected on her second day of hospital stay were positive for SARS-CoV-2 nucleic acid, while her breast milk samples collected on days two, nine, 16, and 19 after delivery were negative. In addition to the majority of evidence that demonstrated negative results, a small proportion of studies showed SARS-CoV-2 positive results in breast milk samples, including studies in Turkey [23, 33], Italy [19, 24, 34, 40], the USA [35], Mexico [42], Australia [53], India [54] and China [56].

### The infectivity of breast milk from infected mothers

A study in the USA [35] reported that one breast milk sample from an infected mother tested positive for SARS-CoV-2 RNA. However, no replication-competent virus was detectable in this positive breast milk sample, which indicated that SARS-CoV-2 in breast milk may not be infectious. No additional studies that directly tested viral infectivity in breast milk were retrieved.

Four studies included in this review focused on the activity of the added SARS-CoV-2 in breast milk after pasteurization. Chambers et al. [35] added SARS-CoV-2 virus into breast milk and then used Holder pasteurization to pasteurize some of the samples. The authors failed to detect SARS-CoV-2 viral RNA or culturable virus in breast milk samples that underwent Holder pasteurization. In contrast, the nonpasteurization samples were found to be positive for viral RNA. A study in Canada [60] added SARS-CoV-2 to breast milk samples from a milk bank and then pasteurized these samples. The study showed that Holder pasteurization of human milk could inactivate SARS-CoV-2 [60]. Similarly, an experiment in Australia [61] demonstrated that Holder pasteurization could inactivate replicative SARS-CoV-2, which was added to breast milk samples from healthy donors. Moreover, Conzelmann et al. [62] added SARS-CoV-2 into five breast milk samples. After pasteurization, no RNA particles were detected in these samples.

### Other transmission modalities associated with breastfeeding

According to the mechanisms of SARS-CoV-2 transmission, the ways of mother-to-child transmission associated with breastfeeding may include close contact transmission and droplet transmission [63]. Tam et al. [53] believed that the risk of environmental and patient’s own oropharynx contamination of breast milk was possible. Recent studies found that the external surfaces of breast milk containers could be contaminated by SARS-CoV-2. Kampf et al. [64] and van Doremalen et al. [65] reported that SARS-CoV-2 was more stable on plastic surfaces (i.e., contamination lasting 2–9 days) and glass surfaces (i.e., contamination lasting 4–5 days). These bottles could be potential sources of contamination and transmission.

### The possible protective effect of breast milk from infected mothers

Nine studies included in this review showed that breast milk from infected mothers contained SARS-CoV-2-specific antibodies, which may be protective for children (Table 3). A study in Brazil detected IgA in a SARS-CoV-2-infected mother’s breast milk [68]. In the USA, all breast milk samples from 18 infected women were reported to contain anti-SARS-CoV-2 IgA and IgG [49]. Another study in the USA [67] detected breast milk samples from eight COVID-19-recovered and seven COVID-19-suspected women 3–4 weeks after symptoms had abated and found that 80% of samples contained IgA and 67% of samples contained IgG and/or IgM binding to the receptor-binding domain [67]. A study in the Netherlands reported that 83% of the confirmed cases and 67% of the suspected cases had SARS-CoV-2 antibodies in their breast milk samples [69]. A study in China [22] also detected anti-SARS-CoV-2 IgG and IgM in breast milk samples of 14 infected mothers [22]. Another study in China detected IgM in 21 out of 38 breast milk samples of infected mothers [50]. In addition, in the study of Fenizia et al. [40], anti-SARS-CoV-2 IgM was detected in breast milk from one confirmed mother. According to a case report [58] in China, anti-SARS-CoV-2 IgG was found in breast milk samples. Finally, COVID-19-positive mothers had breast milk antibodies against the S2 subunit SARS-CoV-2 [66]. The majority of studies did not specify the value of antibody titers. Instead, some studies used graphs to show that antibody titers of milk samples from infected mothers were higher than those from control cases, while others studies stated this phenomenon in brief.

**Table 3:** Summary of evidence about antibodies

Authors	Study country	Sample size	Milk samples	Time of breast milk collection	Detection results	Titers	Detection methods	Infection status
Demers-Mathieu et al. [66]	USA	27	27	Collected during lactation time between 4 and 10 months	S2 SARS-CoV-2-specific IgG level was higher in the COVID-19 group than in the control group	NA	ELISA	7 confirmed, 20 suspected
Fenizia et al. [40]	Italy	10	10	Collected 5 days after delivery, tested SARS-CoV-2 positive during the third trimester	One sample positive for IgM	NA	CLIA	Confirmed, symptomatic
Fox et al. [67]	USA	15	15	3–4 weeks after symptoms abated	80% positive for IgA and 67% positive for IgG and/or IgM	All endpoint titers significantly higher than control samples, 10–10^4^	ELISA	8 confirmed, recovered and 7 suspected
Gao et al. [22]	China	14	14	Collected within 7 days after delivery	3 positive for IgG or IgM	IgG: 103.15–145.31 AU/ml IgM: 19.86–92.01 AU/ml	CLIA	Confirmed, symptomatic
Lebrao et al. [68]	Brazil	1	2	On the 3rd day after delivery and the 6th day since the onset of symptoms	Both positive for IgA	2.5 (3rd day after delivery) and 1.9 (the 6th day since the onset of symptoms)	ELISA	Confirmed, symptomatic
Pace et al. [49]	USA	18	37	12.0 ± 8.9 days after the onset of symptoms	76% positive for IgA and 80% positive for IgG	Antibody concentration of samples from infected mothers higher than antibody concentration of milk samples collected before the pandemic	ELISA	Confirmed, 15 symptomatic and 3 asymptomatic
Peng et al. [50]	China	15	38	Collected at 10 time points: the day of delivery, the 3rd, 7th, 14th, 21st, 28th, 35th, 42nd, 56th and 70th day after delivery	21 positive for IgM	0.1–3.03	ELISA	Confirmed, symptomatic
van Keulen et al. [69]	Netherlands	38	38	Approximately 6 days after the onset of symptoms	83% confirmed cases and 67% suspected cases positive for IgA	NA	ELISA	29 confirmed and 9 suspected
Yu et al. [58]	China	1	2	Collected on the 11th and 27th after the onset of symptoms	Both positive for IgG	NA	ELISA	Confirmed, symptomatic

*CLIA* Chemiluminescence immunoassay

*ELISA* Enzyme-linked immunosorbent assay

### Current recommendations about breastfeeding

National and international organizations have provided different recommendations about breastfeeding during the COVID-19 pandemic [8, 10, 12–16, 70–75], which are described in detail in Table 4. In February 2020, the National Health Commission of the People’s Republic of China recommended stopping breastfeeding for mothers who were suspected/confirmed to have COVID-19 or had not recovered after diagnosis [14]. If the nucleic acid test of the suspected infected mother was negative twice in a row, the newborn could be transferred out of the isolation and observation area and breastfed [14, 73]. In March 2020, ABM [13] suggested breastfeeding among infected mothers with recommendations in home and hospital settings. At home, confirmed mothers should remain separate from other family members, including the infant, except for the occasions of breastfeeding. In the hospital, if the mother was suspected or confirmed to have COVID-19, it was still reasonable to breastfeed or to provide expressed milk for her infant. In April 2020, ISS [10] recommended breastfeeding in a conservative manner. They suggested that asymptomatic or mildly affected mothers consider breastfeeding and rooming-in in coordination with healthcare providers. Separation with attempts to express breast milk to maintain milk production was recommended for severely or critically ill patients.

**Table 4:** International organizations’ recommendations towards breastfeeding during the COVID-19 pandemic

Timeline	International organizations	Recommendations	Specific precautions
Feb 8, 2020	National Health Commission of the People’s Republic of China^a^ [14, 73]	• For mothers who are suspected or confirmed with COVID-19 or have not recovered after diagnosis, breastfeeding should be stopped. If the nucleic acid test of the suspected infected mother is negative twice in a row, the newborn can be transferred out of the isolation and observation area and be breastfed.	• Not available
March 10, 2020	Academy of Breastfeeding Medicine, ABM^a^ [13]	• At home, mothers with confirmed COVID-19 infection should remain separate (home isolation precautions) from other family members and friends or neighbors including the infant, except for breastfeeding. • In hospital, if the mother is well and has only been exposed or is a person-underinvestigation with mild symptoms, breastfeeding with careful precautions is a very reasonable choice. If the mother has COVID-19, it is still reasonable to choose to breastfeed and provide expressed milk for her infant.	• Precautions for breastfeeding directly at the breast. ✓ Washing her hands before touching the infant. ✓ Wearing a face mask. • Precautions for expressing breast milk. ✓ Washing hands before touching any pump or bottle parts. ✓ Following recommendations for proper pump cleaning after each use. ✓ If possible, considering having someone who is well care for and feed the expressed breast milk to the infant. • In the hospital, rooming-in (mother and infant stay in the same room without any other patients in that room) with the infant should keep in a bassinet 6 ft from the mother’s bed. Ideally, there should be another well adult who cares for the infant in the room.
Apr 26, 2020	Italian National Institute of Health, ISS^a^ [10]	• If the mother is severely or critically ill, separation appears to be the best option, with attempts to express breast milk in order to maintain milk production. • If the mother is asymptomatic or mildly affected, breastfeeding and rooming-in can be considered by the mother in coordination with healthcare providers.	• Precautions for breastfeeding directly at the breast. ✓ Cleaning hands. ✓ Using a face mask.
Jun 1, 2020	International Society of Ultrasound in Obstetrics and Gynecology, ISUOG ^b^ [76]	• If the mother is severely or critically ill, separation appears to be the best option, with attempts to express breast milk in order to maintain milk production. • If the mother is asymptomatic or mildly affected, breastfeeding and colocation (also called rooming-in) can be considered by the mother in coordination with healthcare providers, or may be necessary if facility limitations prevent mother-infant separation.	• Precautions for breastfeeding directly at the breast. ✓ Washing hands. ✓ Wearing a three-ply surgical mask before touching the infant. • Precautions for expressing breast milk. ✓ A dedicated breast pump should be used. ✓ The machine should be washed thoroughly, according to the manufacturer’s recommendations, after each use. • In case of rooming-in, the infant’s cot should be kept at least 2 m from the mother’s bed, and a physical barrier such as a curtain may be used.
Jan 25, 2021	World Health Organization, WHO^c^ [8, 77, 78]	• Mothers with suspected or confirmed COVID-19 should be encouraged to initiate or continue to breastfeed • If suspected or confirmed infected mothers are well enough, they should keep skin-to-skin contact with their babies and breastfeed with appropriate precautions. Mothers with symptoms of COVID-19 are advised to wear a medical mask, but even if this is not possible, breastfeeding should be continued. For those who are too unwell to breastfeed, expressing milk and donor human milk could be considered.	• Precautions for breastfeeding directly at the breast. ✓ Washing hands frequently with soap and water or using alcohol-based hand rub and especially before touching the infant. ✓ Wearing a medical mask during any contact with the infant, including while feeding. ✓ Sneezing or coughing into a tissue, then disposing of it immediately and washing hands again. ✓ Routinely cleaning and disinfecting surfaces that mothers have touched.
March 29, 2021	American Academy of Pediatrics, AAP^d^ [75]	• The AAP strongly supports breastfeeding as the best choice for infant feeding. • Counsel families to consider delaying weaning and extending the duration of breastfeeding to maximize the protection conferred via human milk during the pandemic. • If mothers choose not to breastfeed during the first week postpartum, pediatricians should consider asking family whether they might reconsider this choice, and engage in a discussion about the importance of breastfeeding and expressed human milk in protecting against infections and other diseases during this most vulnerable time.	• Precautions for breastfeeding directly at the breast. ✓ Proper hand washing with soap and water before handling the infant. ✓ Wearing a mask. ✓ When not nursing, the infant can be cared for by a healthy caregiver, if available, and/or maintained in a separate room or at least 6 ft away from the mother. • Precautions for expressing breast milk. ✓ Wearing a mask. ✓ Thoroughly cleaning her hands as well as any pump parts, bottles, and artificial nipples. ✓ The expressed milk can be fed to the infant by a healthy caregiver.
June 17, 2021	Centers for Disease Control and Prevention, CDC^d^ [70–72]	• Breast milk is the best source of nutrition for most infants, and it provides protection against many illnesses. There are rare exceptions when breastfeeding or feeding expressed breast milk is not recommended. • People without suspected or confirmed COVID-19 and who have not been in close contact with someone who has COVID-19, or who have been fully vaccinated for COVID-19 do not need to take special precautions when feeding at the breast or expressing milk. • When a lactating caregiver’s milk is not available, pasteurized donor human milk is important for preterm infants. If hospitals have difficulty acquiring donor human milk, available supplies should be prioritized for preterm infants who will benefit most from breast milk.	• Precautions for breastfeeding directly at the breast. ✓ Wearing a mask when they are less than 6 ft from the child during feeding. ✓ Washing hands with soap and water for 20 s before each feeding. • Precautions for expressing breast milk. ✓ A dedicated breast pump should be used. ✓ Wearing a mask when they are less than 6 ft from the child during expression and wash hands with soap and water for 20 s before touching any pump or bottle parts and before expressing breast milk. ✓ Following recommendations for proper pump cleaning after each use. Clean all parts that come into contact with breast milk. ✓ Consider having a healthy caregiver who does not have COVID-19, is not at increased risk for severe illness from COVID-19, and is living in the same home feed the expressed breast milk to the baby. If the caregiver is living in the same home or has been in close contact with you, they might have been exposed. Any caregiver feeding the baby should wear a mask when caring for the baby for the entire time you are in isolation and during their own quarantine period after you complete isolation. • In the hospital, engineering controls like physical barriers are used (e.g., placing the neonate in a temperature-controlled isolator), and the neonate is kept ≥6 ft away from the mother as much as possible. • In a workplace with a multiuser lactation room, efforts should be made to implement engineering and administrative controls to enable physical distancing (e.g., spacing lactation stations at least 6 ft apart, installing physical shields between lactation stations, staggering lactation schedules, encouraging telework).
Jul 29, 2021	United Nations International Children’s Emergency Fund, UNICEF^d^ [16, 74]	• For suspected or confirmed infected mothers who are well enough to breastfeed, breastfeeding should be continued with appropriate precautions. • For those who are too unwell to breastfeed, expressing milk and donor human milk could be considered.	• Precautions for breastfeeding directly at the breast. ✓ Wearing a mask if available. ✓ Washing hands before and after contact. ✓ Cleaning/disinfecting surfaces. • Express milk should be given to infant via a clean cup and/or spoon – all while following the same precautions.
Nov 2 2021	Royal College of Obstetricians and Gynecologists, RCOG^e^ [15]	• Breastfeeding should be recommended to all women in line with usual guidance. • Women with suspected or confirmed COVID-19 should remain with their baby and be supported to practice skin-to-skin/kangaroo care, if the newborn does not require additional medical care at this time. • Adopt a precautionary approach for a woman who has suspected or confirmed COVID-19 and whose baby needs to be cared for on the neonatal unit to minimize any risk of women-to-infant transmission; at the same time, involve parents in decisions, mitigating potential problems for the baby’s health and wellbeing and for breastfeeding, bonding and attachment. • Women and their families should be informed that infection with COVID-19 is not a contraindication to breastfeeding. Women should be supported to make an informed decision about how they feed their baby. Women who choose to breastfeed should be supported to do so, even if they have probable or confirmed COVID-19. • When a woman is not well enough to care for her own infant or where direct breastfeeding is not possible, support her to express her breast milk by hand or using a breast pump, and/or offer access to donor breast milk.	• Precautions for breastfeeding directly at the breast. ✓ Washing hands before touching the infant. ✓ Trying to avoid coughing or sneezing on the infant while feeding at the breast. ✓ Considering wearing a face mask while breastfeeding, if available. • Precautions for expressing breast milk. ✓ Washing hands before touching breast pump or bottles. ✓ Following recommendations for pump cleaning after each use. ✓ Considering asking someone who is well to feed expressed breast milk to the infant. • If mothers are expressing breast milk in hospital, a dedicated breast pump should be used.

^a^No updates were found

^b^The earlier version of the ISUOG’s Guidance [12] was published on Mar 20, 2020. There is no change in breastfeeding recommendations between the earlier version and the updated version

^c^The earlier version of the WHO Guidance [79] was published on May 27, 2020. There is no change in breastfeeding recommendations between the earlier version and the updated version

^d^This is the updated version. The earlier version was not available

^e^The current breastfeeding recommendation was added in version 11 published on July 24, 2020 [15], with no changes since then

With much new evidence emerging in 2021, ISUOG [12, 76], WHO [8, 77, 78], AAP [75], CDC [70–72], UNICEF [16, 74], and RCOG [15] continued updating their breastfeeding recommendations. According to the current evidence, breast milk was unlikely to be the source of transmission of SARS-COV-2 [11, 67, 80, 81]; these organizations consistently recommended that mothers continue to breastfeed their infants with precautions if suspected or known to have COVID-19. The AAP [75] strongly supported breastfeeding as the best choice for infant feeding. The RCOG [15], UNICEF [16, 74], and WHO [8, 77, 78] suggested that suspected or infected mothers stay together with their infants after delivery, keep skin-to-skin contacts and breastfeed directly with careful precautions if mothers feel well. When a woman was not well enough to care for her infant or when direct breastfeeding was not possible, expressing breast milk could be considered. Pasteurized donor human milk was also recommended when mothers’ breast milk was not available by the WHO [8, 77, 78], CDC [70–72], UNICEF [16, 74], and RCOG [15]. In contrast, the ISUOG [76] recommended breastfeeding in a conservative manner: whether to start breastfeeding should be decided by shared decision with the parents with consideration of healthcare providers’ advice.

Precautions for direct breastfeeding put forwards by the above organizations included washing hands before touching the infant, wearing a medical mask during any contacts with the infant, and routinely cleaning and disinfecting surfaces that mothers had touched. Precautions for expressing breast milk included wearing a mask during expression, washing hands before touching any pumps/bottle parts and expressing breast milk, following recommendations for proper pump cleaning after each use, and feeding expressed milk to the infant by a healthy caregiver who was not at risk for COVID-19, if possible.

## Discussion

The present review article summarizes the latest evidence about the safety of breastfeeding and the current recommendations on breastfeeding during the COVID-19 pandemic. The evidence summarization was generally based on van de Perre’s framework, which has been specifically used to assess the likelihood of viral infections during breastfeeding [17]. Consistent with van de Perre et al. [17], we did not find any animal models related to SARS-CoV-2 transmission by breastfeeding. However, we found some evidence related to antibodies in breast milk samples and categorized them into the perspective of “the possible protective effect of breast milk from infected mothers”.

From the perspective of infants’ health status, current evidence suggests that infants breastfed by infected mothers might test positive for SARS-CoV-2; however, the population of infected infants was small. Our findings were consistent with a systematic review [11] that summarized evidence from 17 countries. The systematic review demonstrated that among 148 infants who were breastfed by infected mothers, only seven (4.9%) were infected; in comparison, 5.3% of formula-fed infants were infected. Similarly, a meta-analysis of 176 published cases reported that breastfeeding might not be associated with SARS-CoV-2 infections, and SARS-CoV-2 viral transmission through breast milk might be rare [82]. However, there were studies [18] demonstrating positive results for SARS-CoV-2 among breastfed infants; therefore, vertical and respiratory transmission could not be ruled out. Moreover, the majority of the studies assessed infants at a single time point. Further studies to follow up the health status of breastfed infants are warranted to detect false positive results.

Of studies testing SARS-CoV-2 in breast milk, 66.7% (22/33) reported that milk samples from infected mothers were all negative, and 33.3% reported positive results. The reasons for positive results in some breast milk samples remain unclear. Bastug et al. [33] suggested that the viral load in breast milk, the testing method, the timing of sample collection, and the transport and storage of samples were potential contributors to the positive results. Therefore, the role of breast milk as a vehicle to transmit COVID-19 from mother to newborn could not be confirmed [83]. Further studies are needed to collect breast milk samples from more cases in different regions and at multiple time points.

Few studies have directly tested the viral activity and infectivity of breast milk from infected mothers. Therefore, we could not confirm that SARS-CoV-2 in breast milk was infectious. Studies have focused on the activity of the added SARS-CoV-2 in breast milk after pasteurization. Pasteurization is an important method to eliminate viral and bacterial agents and ensure the safety of donated breast milk in human milk banks [84]. Evidence included in our review suggested that pasteurized breast milk was an alternative and effective option for SARS-CoV-2-infected mothers who were not able to breastfeed directly [26]. However, considering that some of the bioactive components in breast milk could be lost after pasteurization [84], pasteurized breast milk may not be the best choice.

Blackshaw et al. [85] listed infant feeding pathways and possible transmission modalities in their review. Our results were consistent with Blackshaw et al. [85], who found that transmission routes other than breast milk could not be ruled out during feeding. The potential routes included mother and other family members’ skin, bottle surfaces, etc.

This review included evidence showing that breast milk from infected mothers contained SARS-CoV-2-specific antibodies, which may be protective for children. This evidence supported Davanzo et al.’s hypothesis that specific antibodies of SARS-CoV-2 could be passed from the COVID-19-infected mother via breast milk to her infant within a few days after the onset of the disease and modulate the clinical expression of the infant’s infection [10]. In addition, a previous review on vaccination and breastfeeding showed that anti-SARS-CoV-2 immunoglobulins may be transferred from healthy vaccinated lactating mothers to newborns through breastfeeding [86]. In addition to SARS-CoV-2-specific antibodies, as confirmed in the literature, breast milk contains immunoglobulins that can protect infants from a variety of respiratory and digestive infections [87]. For example, whey protein in human milk could block SARS-CoV-2 and its related pangolin coronavirus (GX_P2V) attachment and replication at entry and even post entry to inhibit the virus [88]. Lactoferrin in breast milk can prevent viral infections and may protect infants and boost their innate immune system against COVID-19 [89, 90]. As a result, not breastfeeding could expose infants to a high risk of infections during the COVID-19 pandemic [10].

According to our results and the analytical framework, only two criteria (“viral infection in children breastfed by infected mothers”, and “the presence of virus/antigen/genome in the breast milk of infected mothers”) were met with limited evidence demonstrating positive results. Therefore, we believe that the breast milk of infected mothers is unlikely to transmit SARS-CoV-2. Moreover, a large amount of evidence was from case reports; studies with more robust designs are still lacking. Publication bias might also exist. We thus consider that the safety of breastfeeding during COVID-19 was insufficiently documented. Further studies to follow up the health status of infants who were directly breastfed by their confirmed/suspected infected mothers, to collect breast milk samples at multiple time points for viral tests and to examine specific antibodies in breast milk samples are warranted.

We found that recommendations on breastfeeding during COVID-19 from different organizations were controversial. Similarly, a previous review found that the aspects in the guidance documents from 33 countries were poorly consistent with the WHO guidelines [91]. The other study found that in 73 articles, recommendations regarding breastfeeding, separation of mother and newborns after birth and decontamination practices were varied [92]. In our review, China seemed to have stricter measures about breastfeeding. Chinese experts thought that breastfeeding should be stopped among confirmed or suspected mothers, as the possibility of the vertical transmission of COVID-19 could not be completely ruled out [93, 94]. The ISS and ISUOG also recommended breastfeeding in a conservative manner. They thought decisions towards breastfeeding should be made by mothers and their family members, with the consideration of healthcare providers’ advice [10, 76]. In comparison, other organizations (e.g., ABM, WHO, AAP, CDC, UNICEF, and RCOG) encouraged breastfeeding among suspected or confirmed infected mothers. Indeed, the currently available scientific evidence does not allow us to accurately inform the best practices of breastfeeding during the COVID-19 pandemic [80]. As new evidence accumulates, recommendations should be updated in a timely manner to ensure appropriate practices.

## Conclusions

This article provides comprehensive evidence for the safety and recommendations of breastfeeding during the COVID-19 pandemic. Based on an analytical framework, the current evidence proved that transmission of SARS-CoV-2 from infected mothers via breast milk was unlikely to happen. However, owing to the low quality of the current evidence, the safety of breastfeeding during COVID-19 is still insufficiently reported. Further studies with robust designs are warranted to determine the safety of breastfeeding. Studies to follow up the health status of infants who were breastfed by confirmed/suspected infected mothers, to conduct viral tests on breast milk samples at multiple time points and to examine specific antibodies in breast milk samples are needed to fill the research gaps. This review contributed to the literature by providing scientific evidence and recommendations on breastfeeding and identifying the focus for further research during the COVID-19 pandemic.

## Data Availability

Not applicable.

## Abbreviations

AAP: American Academy of Pediatrics
ABM: Academy of Breastfeeding Medicine
CDC: U.S. Centers for Disease Control and Prevention
CNKI: China National Knowledge Infrastructure
COVID-19: Coronavirus Disease 2019
ISS: Italian National Institute of Health
ISUOG: International Society of Ultrasound in Obstetrics and Gynecology
RCOG: Royal College of Obstetricians and Gynecologists
SARS-CoV-2: Severe Acute Respiratory Syndrome Coronavirus 2
UNICEF: United Nations International Children’s Emergency Fund
WHO: World Health Organization
